# The Cytosolic Chaperonin CCT/TRiC and Cancer Cell Proliferation

**DOI:** 10.1371/journal.pone.0060895

**Published:** 2013-04-16

**Authors:** Chafika Boudiaf-Benmammar, Thierry Cresteil, Ronald Melki

**Affiliations:** 1 Laboratoire d’Enzymologie et Biochimie Structurales, CNRS, Gif-sur-Yvette, France; 2 Institut de Chimie des Substances Naturelles, CNRS, Gif-sur-Yvette, France; Bambino Gesù Children's Hospital, Italy

## Abstract

The molecular chaperone CCT/TRiC plays a central role in maintaining cellular proteostasis as it mediates the folding of the major cytoskeletal proteins tubulins and actins. CCT/TRiC is also involved in the oncoprotein cyclin E, the Von Hippel-Lindau tumour suppressor protein, cyclin B and p21^ras^ folding which strongly suggests that it is involved in cell proliferation and tumor genesis. To assess the involvement of CCT/TRiC in tumor genesis, we quantified its expression levels and activity in 18 cancer, one non-cancer human cell lines and a non-cancer human liver. We show that the expression levels of CCT/TRiC in cancer cell lines are higher than that in normal cells. However, CCT/TRiC activity does not always correlate with its expression levels. We therefore documented the expression levels of CCT/TRiC modulators and partners PhLP3, Hop/P60, prefoldin and Hsc/Hsp70. Our analysis reveals a functional interplay between molecular chaperones that might account for a precise modulation of CCT/TRiC activity in cell proliferation through changes in the cellular levels of prefoldin and/or Hsc/p70 and CCT/TRiC client protein availability. Our observation and approaches bring novel insights in the role of CCT/TRiC-mediated protein folding machinery in cancer cell development.

## Introduction

To ensure efficient folding of nascent polypeptide chains in a highly crowded environment cells have designed a class of proteins known as molecular chaperones [1], [2]. These proteins bind, during or after translation, unfolded, partially folded and misfolded polypeptide chains, often through exposed hydrophobic segments [3]. Binding of molecular chaperones to their clients counteracts their intrinsic aggregation propensity and allows a polypeptide chain to search the folding landscape and reach its native, functional state [4]. Molecular chaperones also control protein homeostasis under normal and stress conditions. They constitute therefore a quality control system for the maintenance of native protein conformation, translocation of proteins across membranes and normal protein turnover [2].

The involvement of molecular chaperones in cancer development and progression is subject to active debate. Several studies report that chaperones are found at increased levels in many solid tumours and haematological malignancies [5], [6]. Their expression may in part account for the ability of malignant cells to maintain protein homeostasis in the unfavourable hypoxic and acidic microenvironment of the tumour. Through their interaction with key regulatory proteins, molecular chaperones regulate the cell cycle and protect the cells from programmed death. They promote tumour cell survival, growth and metastasis, even in growth factor deprived conditions, by allowing continued protein translation and cellular proliferation [7]. Finally, molecular chaperones are considered critical for allowing tumour cells to tolerate genetic alterations that would otherwise be fatal [5]. Indeed, molecular chaperones such as Hsp90 act as biochemical buffers for the numerous genetic lesions that are characteristic of most human cancers and drives oncogenesis [8].

Molecular chaperones are ubiquitous proteins that are the products of distinct, highly conserved, gene families. They are classified into different categories based on their molecular masses, cellular distribution and function [9]. The Hsp60 family members are peculiar in that they form high molecular weight ring-shaped protein complexes. These particles are true folding nanomachines fuelled by ATP and termed chaperonins. Two classes of chaperonins have been defined [10]. The chaperonins constituting group I are constituted by a single polypeptide chain and have a 7 fold symmetry. This group comprises GroEL [11] and its mitochondrial counterpart cpn60. The chaperonins constituting group II have an 8 fold symmetry and comprise archaebacterial thermosomes and the cytosolic chaperonin contaning t-complex polypeptide 1 (CCT) also known as the TCP1 ring complex (TRiC) [12]; CCT/TRiC is a 16 subunits complex composed of two back-to-back stacked rings, each containing eight different subunits of approximately 60 kDa (α, β, γ, δ, ε, ζ−1, η and θ) [13]; [14]. CCT/TriC cooperates with protein cofactors to fold target client proteins. Hop/p60, a cofactor of Hsp70 and Hsp90, increases folding efficiency by facilitating nucleotide exchange [15]. Phosducin like protein 3 (PhLP3) is a negative modulator of folding and restrains client protein access to the folding chamber [16]. Finally the molecular chaperone prefoldin (PFD) also modulates CCT/TRiC activity as it delivers client proteins [17], [18].

CCT/TRiC mediates the folding of tubulins and actins [19]; [20] including the centrosomal γ-tubulin and centractin [21]. The growing list of CCT/TRiC clients comprises proteins involved in tumor genesis with cyclin E [22], the Von Hippel-Lindau (VHL) tumour suppressor protein [23], cyclin B and p21^ras^ [24]. Beside its requirement for actins and tubulins folding, there is evidence that CCT/TRiC is strongly up-regulated during the G1/S phase transition of the cell cycle through to the early S phase, and that this event is controlled at the mRNA level [25]. Moreover, the down regulation of CCTα expression is associated with an inhibition of cell proliferation, a decrease in cell viability, cell cycle arrest and cellular apoptosis [26].

Altogether, these observations strongly suggest that CCT/TRiC plays a key role in cell cycle progression and that it could be implicated in tumour development.

Here we quantify i) the expression levels of CCT/TRiC and its partners including Hsc 70 and Hsp70 (Hsc/p70), PhLP3, Hop/P60, prefoldin and DNAJB1 in cancerous human cell lines and ii) its activity in the cell extracts. Overall, the expression levels of CCT/TRiC in cancer cell lines are higher than that in normal cells. Strikingly however CCT/TRiC activity does not always correlate with its expression levels. We therefore documented the expression levels of CCT/TRiC modulators. Our analysis brings novel insights in the role of protein folding machinery in cancer cell development.

## Materials and Methods

### Human Cell Lines and Cell Extracts Preparation

Fifteen human cancer cell lines, derived from cancers of different organs and showing distinctive characteristics, and a non-cancerous cell line established from normal fetal lung tissue were obtained from the American Type Culture Collection (ATCC; Rockville, MD). Three other cell lines came from other sources: SH-5YSY was purchased from Sigma-Aldrich (Saint Louis, USA), OVCAR-8, an NCI cell line, was provided by Dr Liscovitch (Rehovot, Israel) and SF268 was obtained from the National Cancer Institute (NCI Frederick, MD, USA). MIA-PaCa-2, MRC5-SV2, HepG2 and KB cells were cultured in Dulbecco’s Modified Eagle’s Medium (DMEM) supplemented with 10% fetal bovine serum (FBS). HCT116, SK-OV3, HT-29, K-562, HL-60, PC3, HeLa, MCF-7, MDA-MB-435S, MDA-MB-431, A549, SF-268, HCT15 and OVCAR-8 cells were routinely cultured in 10% FBS/RPMI 1640 supplemented with penicillin-streptomycin, fungizone and glutamine. SH-5YSY cells were cultured in a mixture of DMEM and Ham’s F12 medium supplemented with 10% FBS, glutamine and penicillin-streptomycin. The cell lines characteristics and references are given in Table 1.

**Table 1:** Characteristics of the cell lines and cell extracts used throughout this study.

Number	Designation	Tissue of origin		Total proteins concentration(mg/ml)	Doubling time (hour)
ATCC/CCL-1420	MIA-PaCa-2	pancreas	carcinoma	24	24
ATCC/CCL-247	HCT116		colorectal carcinoma	20	19
ATCC/CCL-225	HCT15	colon	colorectal adenocarcinoma	20	20
ATCC/HTB-38	HT29		colorectal adenocarcinoma	20.6	18
ATCC/HB-8065	HepG2	liver	hepatocellular carcinoma	18	48
NCI/0502763	SF268	brain	astrocytoma	21.5	30
Sigma/94030304	SH-SY5Y	nervous system	neuroblastoma	8	35
ATCC/CRL-1435	PC3	prostate	adenocarcinoma	20	24
ATCC/HTB-77	SK-OV3		adenocarcinoma	21	48
	OVCAR-8	ovary		25	26
ATCC/HTB-26	MDA-MB-231			16	24
ATCC/HTB-22	MCF-7	breast	adenocarcinoma	19	22
ATCC/HTB-129	MDA-MB-435S	skin	melanoma	17	20
ATCC/185	A549		carcinoma	24.5	23
ATCC/171	MRC-5-SV2	lung	normal fibroblast	28	25
ATCC/CCL-240	HL-60	peripheralblood	acuete promyelocytic leukemia	19	18
ATCC/CCL-243	K562	bone marrow	chronic myelogenous leukemia	27	16
ATCC/CCL-2	HeLa		adenocarcinoma	16.5	24
ATCC/CCL-17	KB	cervix	HeLa contaminant	25	19

Cells were counted, washed and harvested. The cell density was adjusted to 24.10^7^ cells/ml in lysis buffer (Tris 10 mM, EDTA 1 mM, pH 7.5, 1 mM phenylmethylsulfonyl fluoride). Suspended cells were placed on ice and subjected to 10 cycles of sonication consisting of a 1 second impulse with an output power of 10 watts spaced by 15 seconds using a Branson sonifier 150 while the human liver fragment (HNCL), which origin and collection prior to 1995 following the ethical committee of the Institut National de la Santé et de la Recherche Médicale are described in [27], [28], was homogenized on ice using a Kontes Teflon-glass homogenizer. Complete cell lysis was checked using a microscope and the ionic strength of the extracts was adjusted to 140 mM Tris/HCl, pH 7.5, 14 mM MgCl_2_, 1 mM DTT, 70 mM KCl, 4 mM ATP and 10% glycerol (v/v). The extracts were clarified by centrifugation at 12000×g for 10 min at 4°C and the protein concentration in the supernatants was determined by the Bradford method [29]. Rabbit reticulocyte lysate (RRL) prepared as previously described [30] was used as a control in all experiments.

### Antibodies

Mouse polyclonal antibodies against human PhLP3 (anti TXNDC9), Hop/p60 (anti STIP1) and PFD5 (anti PFDN5) were purchased from Abnova (Ontario, Canada). Mouse monoclonal antibody against Hsc70/p70 was purchased from StressGen (Victoria, BC, Canada). Rabbit polyclonal antibody to DNAJB1 was purchased from Sigma-Aldrich (Saint Louis, USA). Rabbit polyclonal antibody against CCTα was generated in the laboratory [24].

### Western Blotting

A standard range comprising CCTα (100, 50, 25, 12.5 µg/ml), PhLP3 (0.5, 0.25, 0.125 µg/ml), PFD5 (25, 12.5, 6.25 µg/ml), Hop/p60 (6, 3, 1.5 µg/ml) and Hsp70 (80, 40, 20, 10 µg/ml) was resolved by SDS- polyacrylamide gel electrophoresis (PAGE) using 12% polyacrylamide gels [31] with two dilutions (1/4 or 1/8 and 1/16) of each cell extract and blotted on nitrocellulose filter [32]. Each filter was probed successively with the five primary antibodies after membrane regeneration by incubation at 50°C in 62.5 mM Tris/HCl pH 6.8, 100 mM β-mercaptoethanol and 2% SDS (v/v). Reactive bands were visualized by chemiluminescence using Pierce ECL Western blotting substrate (Thermo scientific, Rockville, USA) following the manufacturer recommendations and the chemiluminescent signal was digitally captured with a LAS-3000 luminescent image analyzer (Fujifilm). All experiments were run at least in duplicate for biological replicates, corresponding to independent cell cultures and mean values and standard deviation were derived from the two independent measurements. The amounts of CCT/TRiC, PhLP3, PFD5 and Hop/p60 in the different extracts were quantified by comparing the luminescence in the cell extracts to that of the standards of known concentration electrophoresed on the same gel.

### Folding Activity Assay

[^35^S]-labeled unfolded β-actin target protein was generated by expression in *E. coli* and purified as described by Gao et al. [33]. The refolding activities of cell extracts, RRL and HNCL were assayed and analyzed following dilution from denaturant of radiolabeled β –actin into cell extracts, RRL and HNCL diluted in folding buffer (80 mM MES/KOH, pH 6.8, 1 mM MgCl_2_, 1 mM DTT, 1 mM EGTA, 1 mM ATP). The protein concentration within the extracts was 15 mg/ml or 2.5 mg/ml. The reaction products were resolved on 6% non-denaturing polyacrylamide gels. The gels were then dried and exposed to a storage phosphor screen (Molecular Dynamics, Sunnyvale, CA, USA) and developed on a Storm PhosphorImager (Molecular Dynamics, Sunnyvale, CA, USA). An example of a CCT-mediated unfolded β-actin refolding reaction is shown in Figure S1. All experiments were run in duplicate. The mean values and standard deviation were derived from the two independent measurements.

### Cell Extracts Immuno-depletion

Two arbitrarily chosen cell extracts (MIA-PaCa-2 and OVCAR-8) and the control were immuno-depleted using either anti- PhLP3, anti-Hop/p60, anti- PFD5, anti-Hsc/p70 or anti-CCTα antibodies. The extracts were incubated for 2 h at 4°C under gentle motion with the adequate antibody and the protein-antibody complexes were sedimented after overnight incubation at 4°C with Protein A Sephrose CL-4B beads (GE Healthcare) equilibrated in 50 mM Tris-HCl, pH 7.0. The supernatants were recovered, the protein concentration was measured and the ability of the immuno-depleted extract to refold [^35^S]-labeled unfolded β-actin was assayed *in vitro*.

### Image Analysis, Statistical Analysis and Calculations

Digital images obtained with the Fujifilm LAS-3000 analyzer and the Storm PhosphorImager were analysed using the software NIH Image (U.S. National Institute of Health, Bethesda, MD).

The intensities derived from image analysis were analyzed using two-sample, one-tailed independent Student’s **t**-tests.

The number of CCT/TRiC and PFD particles per cell was derived from concentration measurements using the following equation:
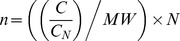
Where n is the number of CCT/TRiC or PFD particles, C, the amount of CCT/TRiC or PFD estimated from western blot measurements by comparison with known amounts of CCTα or PFD5 in mg/ml, C_N_, is the number of cells/ml, Mw, is the molecular weight of CCT/TRiC or PFD particles (750000 for CCT/TRiC, 100670 for PFD) and *N* the constant of Avogadro (6.023×10^23^).

## Results

### CCT/TRiC Expression and Activity

**Figure 1:**
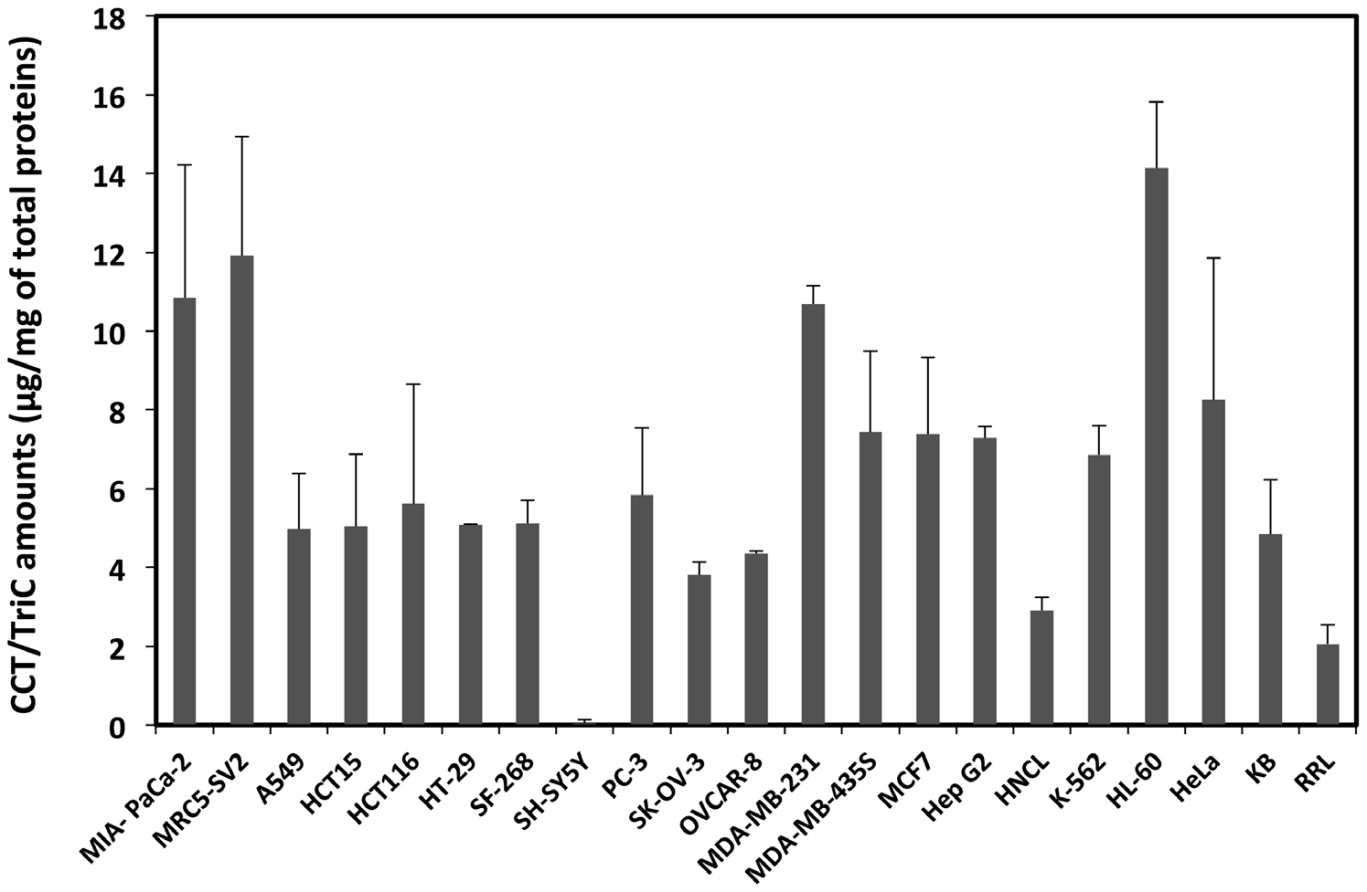
CCT/TRiC quantities in cancer cell lines. The amount of CCT/TRiC in 18 cancer and a normal (MRC5-SV2) cell lines extracts, prepared as described in the experimental procedures section, a non-cancer liver homogenate (HNCL) and rabbit reticulocyte lysate (RRL) was determined by comparing CCTα intensity measured for cell extracts diluted 4 and 16 fold to that of known amounts of CCTα (100, 50, 25, 12.5 µg/ml) migrated on the same 12% SDS polyacrylamide gel by Western blotting. The average amounts of CCT/TRiC in the different extracts are expressed as a function of the total proteins content of the extracts.

**Figure 2:**
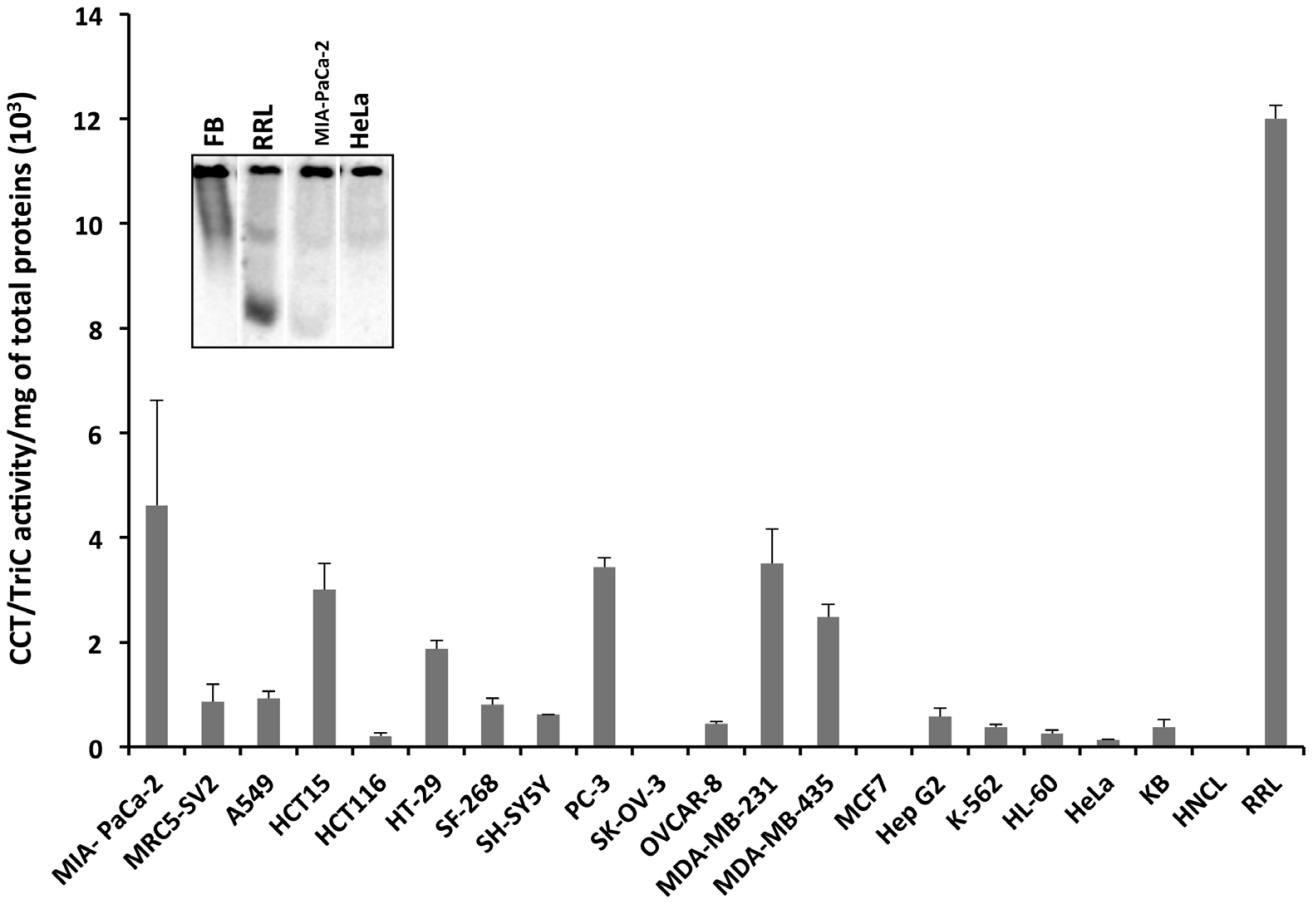
CCT/TRiC folding activity in cancer cell lines extracts. CCT/TRiC folding activity in the 18 cancer cell lines, the normal cell line MRC5-SV2 and the human non-cancerous liver was assayed using [^35^S]-labeled, denatured β-actin folding as described in the experimental procedures section. The reaction products were analyzed on 6% non-denaturing polyacrylamide gels. CCT/TRiC specific activity, derived from the quantification of β-actin bands intensities is expressed as a function of total protein concentration. The images, recorded using a phosphorimager for [^35^S]-labeled actin refolding reactions in folding buffer, RRL, MIA-PaCa-2 and HeLa cell extracts resolved on a native 6% polyacrylamide gel, are shown in the inset.

CCT/TRiC is strictly required for the folding of the two main cytoskeletal proteins actins and tubulins *in vivo*. However, little is known about its expression and activity in cancer cells. We compared the expression levels and activity of CCT/TRiC in 18 cancer cell lines from different organs and a normal cell line (MRC5-SV2), a human non-cancer liver homogenate (HNCL) and rabbit reticulocyte lysate (RRL), where CCT/TRiC is known to be expressed and highly active. The quantification of CCT/TRiC on western blots (Figure 1) revealed that, the average concentration of CCT/TRiC in cancer cell extracts is about 5 µg per mg of total proteins and approximately 3–4.10^8^ CCT/TRiC particles (see experimental procedures section for calculation Table S1 for CCT/TRiC particles number in the different cell lines) except for MIA-PaCa2, MRC5-SV2, MDA-MB-231 and HL-60 cell lines where CCT/TRiC amounts are 2 to 3-fold the average value. In HNCL and RRL, the amount of CCT/TRiC is half that of the average measured for cancer cell lines. It is also worth noting that this amount is the lowest (1.6µg/mg of total proteins) in the human neuroblastoma SH-SY5Y cell line. CCT/TRiC expression thus appears upregulated in all cancer cell lines, in particular, in MIA-PaCa2, MDA-MB-231 and HL-60 cell lines (Figure 1). To determine whether CCT/TRiC expression correlates with its activity we quantified radiolabeled, denatured, actin folding in the cell extracts supplemented with Mg^2+^ and ATP as described in the experimental procedures section. The results, presented in Figure 2 showed that CCT/TRiC activity does not always correlate with its expression level. Indeed, while the folding activity of CCT/TRiC in MIA-PaCa2, MDA-MB-231 and MDA-MB-435S cell line extracts was highly consistent with the important levels of CCT/TRiC within the extracts, it was found very low in HL-60 cell line extract, i.e. the cell line containing the highest amounts of CCT/TRiC, compare Figure 1 to Figure 2.

### Expression of CCT/TRiC Activity Modulators

**Figure 3:**
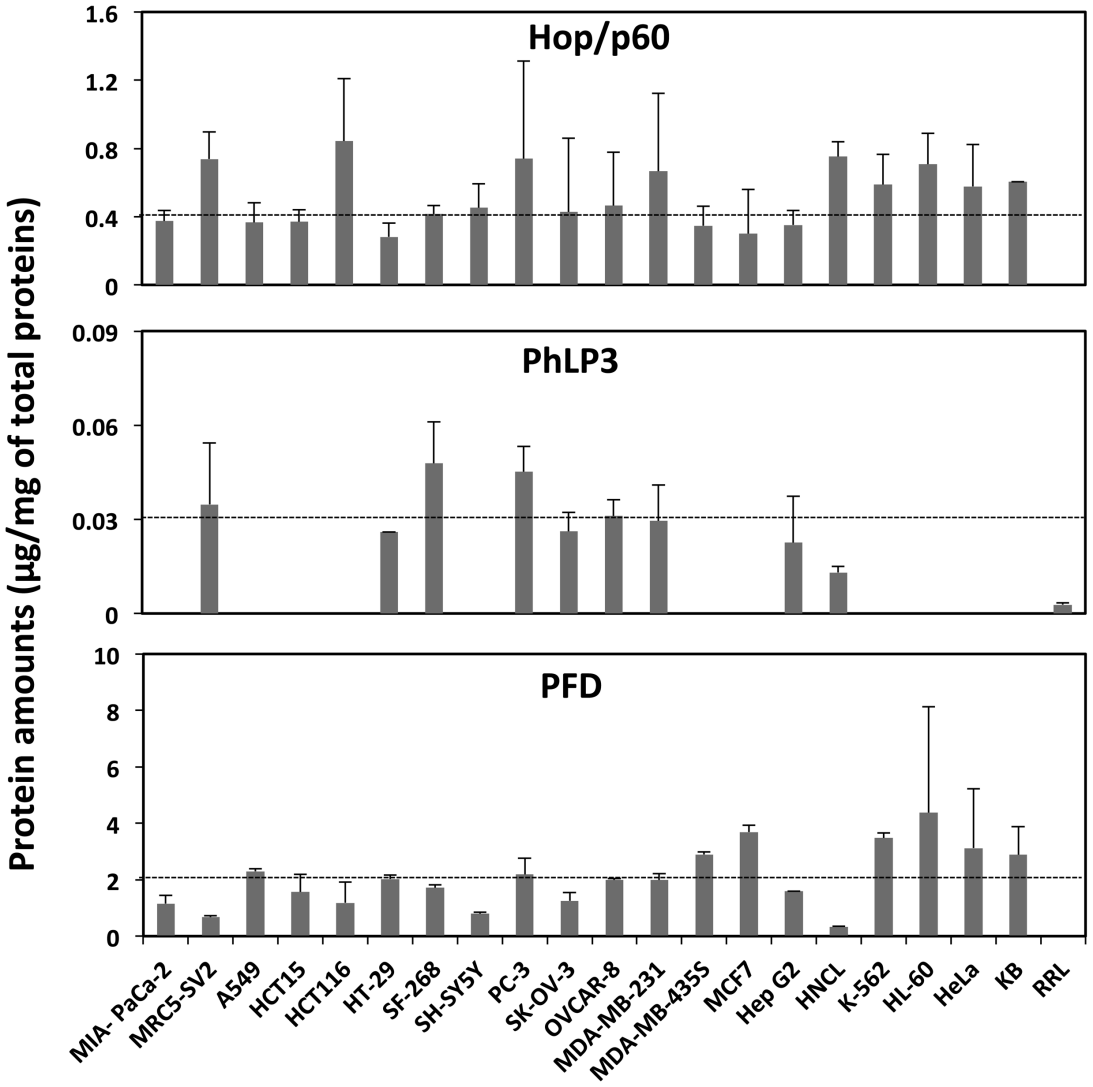
CCT/TriC activity modulators in cancer cell lines extracts. The amount of (**A**) Hop/p60, (**B**) PhLP3 and (**C**) PFD in 18 cancer and a normal (MRC5-SV2) cell lines extracts, a non-cancer liver homogenate (HNCL) and rabbit reticulocyte lysate (RRL) was determined by comparing the chemiluminescence signal recorded for Hop/p60, PhLP3 and PFD in cell extracts diluted 4 and 16 fold to that of known amounts of the proteins on the same Western blots.

As the expression level of CCT/TRiC does not correlate with its activity and to better understand how the latter is modulated in cancer cells, we quantified CCT/TRiC activity modulators, prefoldin, Hop/p60 and PhLP3, in the cell lysates. The data presented in Figure 3A show that Hop/p60 concentration within the cell lines we used is about 0.4 µg/mg of total proteins on average. Hop/p60 appears under-expressed in the majority of the cell lines we used when its expression level is normalized to that in the non-cancer liver homogenate (HNCL) used throughout this study (Figure S2). Hop/p60 expression is the highest in MRC5-SV2, HCT116, PC3 and HL-60 cell lines as it is in HNCL.

CCT/TRiC expression is also high in MRC5-SV2 and HL-60. Surprisingly however, given that Hop/p60 is a nucleotide exchange factor expected to favor CCT/TRiC-mediated folding, the folding activity of MRC5-SV2 and HL-60 cell extracts is low. Similarly, while CCT/TRiC and Hop/p60 concentrations are relatively high in HCT116 cell extracts, the folding activity of the extract is amongst the lowest. We conclude from these observations that the expression level of Hop/p60 is not solely responsible of increased or decreased CCT/TRiC-mediated folding efficiency. We therefore documented PhLP3, the functional antagonist of Hop/p60, expression in the cancer cell lines we used. PhLP3 expression varies significantly from one cell line to another. The protein was undetectable in 11 cancer cell lines we used and of about 0.03 µg/mg of total proteins (Figure 3B) in 8 other cell lines. When detectable, PhLP3 appears over expressed when its expression level is normalized to that in HNCL (Figure S2). Here again, we were not able to correlate a high PhLP3 expression level with a low CCT/TRiC activity. Indeed, PhLP3 concentration is amongst the highest in PC3 and MDA-MB-231 cell extracts (Figure 3B) where CCT/TRiC activity is amongst the highest (Figure 2).

We finally quantified prefoldin expression levels as this co-chaperone assists CCT/TRiC in buffering client protein concentrations in the cytosol and in delivering them to CCT/TRiC. Prefoldin concentration varies little from one cell to another and is on average 2 µg/mg of total proteins (Figure 3C). Prefoldin expression level is twice lower than the average in MIA-PaCa2, MRC5-SV2, HCT116, SH-SY5Y and SK-OV-3 cell extracts and is significantly higher than the average in HL-60, K562, HeLa, KB, MDA-MB-231 and MCF7 cell extracts. Thus a correlation exists between CCT/TRiC activity and the expression level of prefoldin. The highest prefoldin is expressed, the lowest the activity of CCT/TRiC is. Finally, it is worth noting that PFD is expressed to higher levels in all cancer cell lines as compared to HNCL (Figure S2). As this protein is a part of a complex composed of 6 subunits, the average number of active particles per cell ranges from 5 to 24 10^8^ (Table S1).

**Figure 4:**
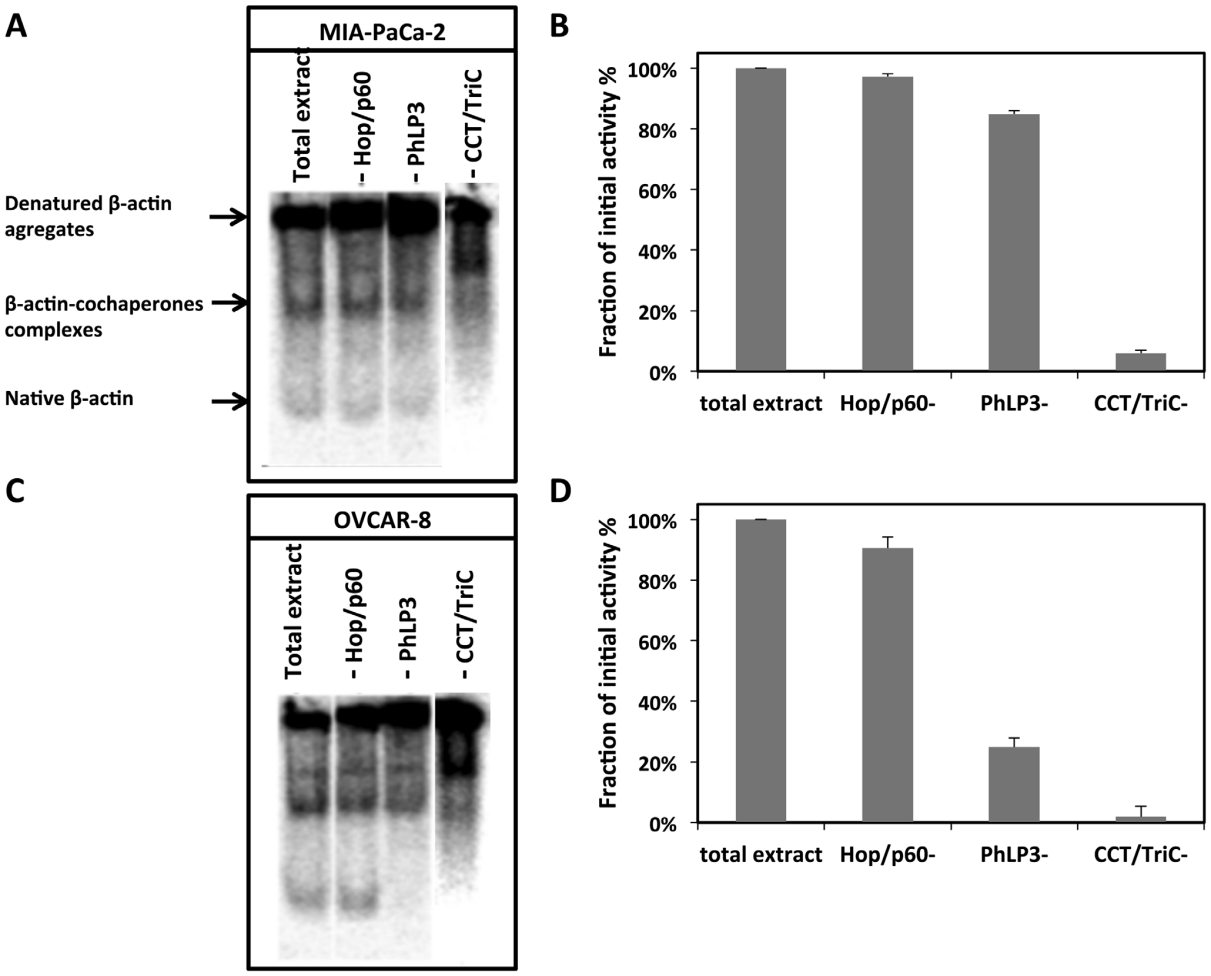
CCT/TriC, Hop/p60 or PhLP3 immunodepletion affects actin refolding. CCT/TRiC folding activity in (**A**) MIA-PaCa-2 and (**C**) OVCAR-8****cell extracts before and after CCT/TriC, Hop/p60 or PhLP3 immunodepletion was assayed using [^35^S]-labeled, denatured β-actin. The protein concentration of the MIA-PaCa-2 and OVCAR-8 cell extracts was 2.5 and 15 mg/ml, respectively. Native β-actin band intensity was measured using a PhosphorImager. The amount of native β-actin measured after immunodepletion is expressed as a fraction of the amount measured for the cell extracts before immunodepletion in (**B**) MIA-PaCa-2 and (**D**) OVCAR-8 cell extracts.

The lack of correlation between increased or decreased expression levels of Hop/p60 and PhLP3 and CCT/TRiC activity suggests that the latter is not efficiently modulated by these polypeptides. To make sure this is indeed the case and better understand how Hop/p60 and PhLP3 affect CCT/TRiC folding activity, we examined the consequences of immuno-depleting Hop/p60 or PhLP3 from two arbitrarily chosen cell extracts: MIA-PaCa-2 (Figure 4A) and OVCAR-8 (Figure 4C) on CCT/TRiC activity. The immuno-depletion of Hop/p60 had no effect on CCT/TRiC activity (Figure 4B, D). In contrast, PhLP3 immuno-depletion from a cell extract where it is significantly expressed, such as OVCAR-8, led to a dramatic decrease in CCT/TRiC activity (Figure 4B, D). This strongly suggests that PhLP3 is heavily involved in modulating CCT/TRiC activity.

### Hsc/p70 Modulate CCT/TRiC Activity

**Figure 5:**
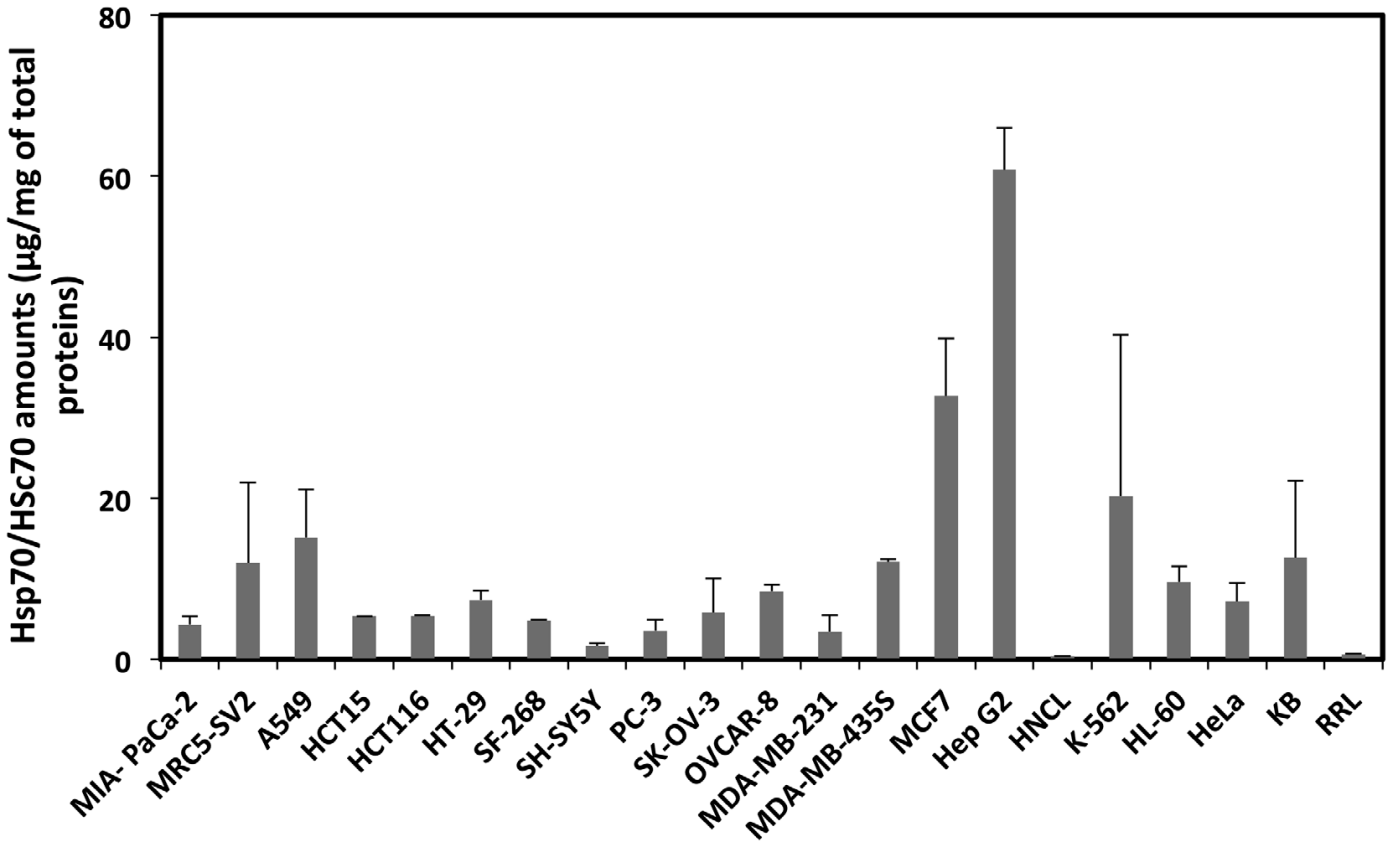
Hsc/p 70 expression in cancer cell lines extracts. The amount of Hsc/p70 in 18 cancer and a normal (MRC5-SV2) cell lines extracts, a liver homogenate (HNCL) and rabbit reticulocyte lysate (RRL) was determined by comparing the chemiluminescence signal recorded for Hsc/p70 in cell extracts diluted 4 and 16 fold to that of known amounts of the protein on the same Western blots. The average amounts of Hsc/p70 in the different extracts are expressed as a function of the total proteins content of the extracts.

CCT/TRiC cooperates with other molecular chaperones [2]. Hsc 70 and Hsp70 (Hsc/p70) have been shown to promote client proteins binding to CCT/TRiC for example [34], [35]. To determine whether the Hsc/p70 expression levels correlate with that of CCT/TRiC activity, the amounts of Hsc/p70 in the cancer cell line extracts we used throughout this study were quantified by Western blotting. The doublet bands observed on the blots originate from the constitutive (Hsc70, 73 kDa) and stress induced form (Hsp70, 72 kDa) of the chaperone. Hsp70 appears overexpressed in the cell lines we used as witnessed by the intensity of the lightest bands on the Western blots. This strongly suggests that the stress-associated form of the chaperone is induced in cancer cell lines. The amount of Hsc/p70 in the different cell extracts we measured is 5 µg/mg of total proteins on average except for MRC5-SV2, A549, K-562, MDA-MB-435S and KB cell lines where Hsc/p70 concentration is 2 to 3-fold higher and SH-5YSY where it is 2 fold lower (Figure 5). Likewise, in the breast adenocarcinoma cell line MCF7 and the hepatocarcinoma cell line HepG2, Hsc/p70 is 7 to 12-fold the average value. Finally, it is worth noting that Hsc/p70 expression is upregulated in all cancer cell lines with expression levels reaching in HepG2 cells 160 fold that within the non-cancer liver homogenate (HNCL) used as a reference in HepG2 cells (Figure 5).

Our results suggest that CCT/TRiC activity is amongst the lowest in cell lines expressing the highest Hsc/p70 levels (compare CCT/TRiC activity to the amounts of Hsc/p70 in MCF7 HepG2 and K-562). Similarly, CCT/TRiC activity appears the highest in cell line extracts where Hsc/p70 levels are lower than average (compare CCT/TRiC activity to the amounts of Hsc/p70 in MIA-PaCa-2, HCT15, PC-3, and MDA-MB-231). This could well be the consequence of client protein partitioning between Hsc/p70 and CCT/TRiC with increased amounts of client proteins unavailable for binding to CCT/TRiC in the presence of high Hsc/p70 concentrations.

**Figure 6:**
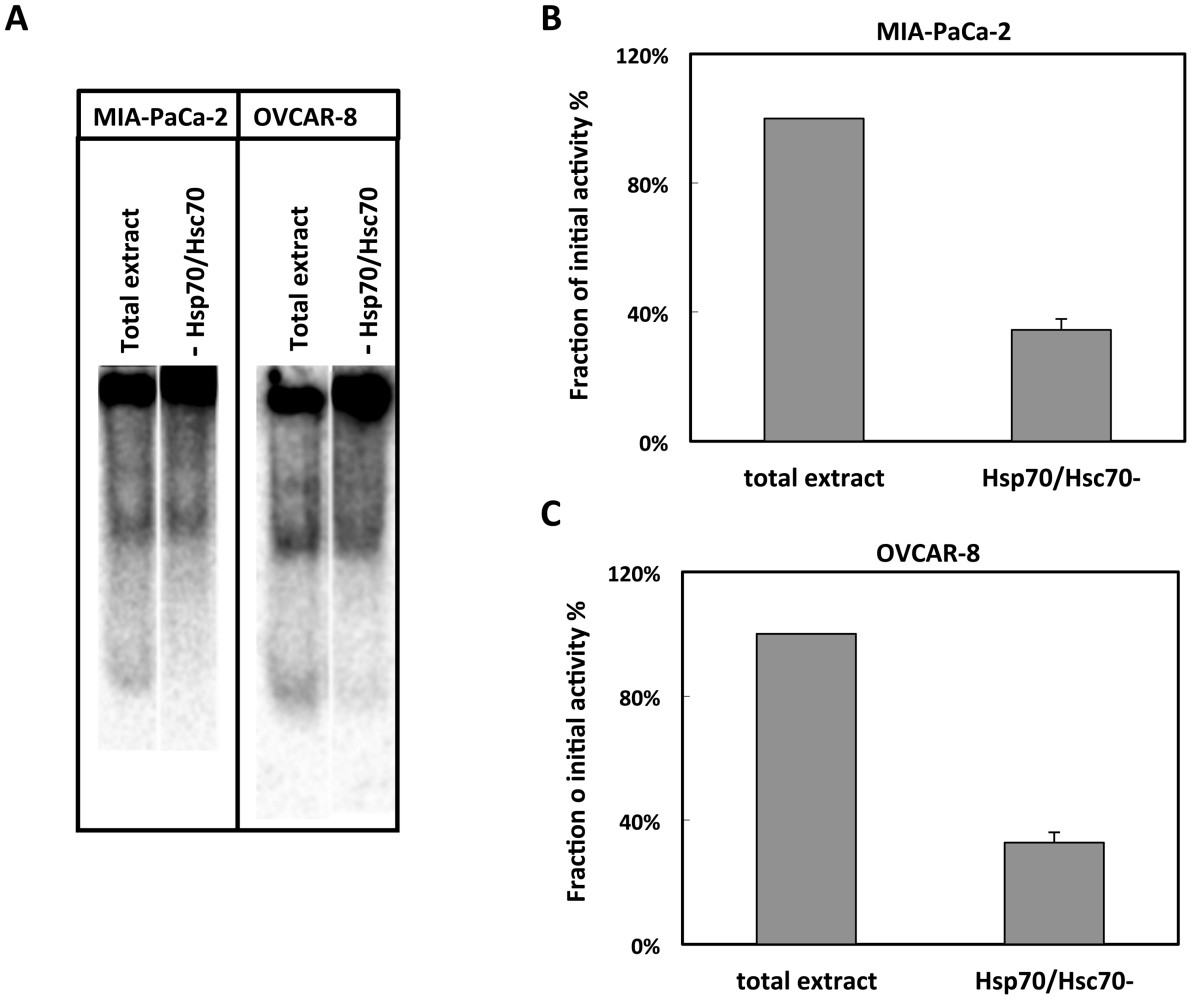
Hsp/c70 immunodepletion affects CCT/TRiC-mediated β-actin folding. CCT/TRiC-mediated labeled, denatured β-actin folding in (**A**) MIA-PaCa-2 and OVCAR-8 cell extracts before and after Hsc/p70 immunodepletion. The protein concentration of the MIA-PaCa-2 and OVCAR-8 cell extracts was 2.5 and 15 mg/ml, respectively. Native β-actin band intensity was recoded using a PhosphorImager. The amount of native β-actin measured after immunodepletion is expressed as a fraction of the amount measured for the cell extracts before immunodepletion in (**B**) MIA-PaCa-2 and (**C**) OVCAR-8 cell extracts.

To further document Hsc/p70 and CCT/TRiC cooperation in protein folding, we assayed CCT/TRiC client protein folding activity in cancer cell extracts following Hsc/p70 immunodepletion. The data presented in Figure 6 clearly shows that CCT/TRiC-mediated β-actin folding decreases by over 65% in MIA-PaCa-2 and OVCAR-8 upon Hsc/p70 immunodepletion. These results clearly demonstrate that Hsc/p70 and CCT/TRiC cooperate.

Our observations are compatible with the view that Hsc/p70 buffers CCT/TRiC client proteins [36]. Indeed, the immunodepletion of Hsc/p70 leads to a reduction of CCT/TRiC client polypeptide pool due to a decrease of the amount of available CCT/TRiC client proteins.

## Discussion

CCT/TRiC plays a central role in cell proliferation through its ability to facilitate the folding of i) - cytoskeletal proteins involved in cell division, e.g. α, β and γ-tubulins [23], [15], [31], [25], α and β actins and centractin [14], [36], [25], ii) - oncoproteins such as cyclin E [22], the Von Hippel-Lindau (VHL) tumour suppressor protein [23], cyclin B and p21^ras^ [24]. CCT/TRiC expression is furthermore up-regulated during the G1/S phase transition of the cell cycle [25] while its down-regulation leads to the inhibition of cell proliferation, a decrease in cell viability, cell cycle arrest and cellular apoptosis [26].

To determine whether CCT/TRiC activity depends simply on its expression level, we compared the expression levels of CCT/TRiC in 18 human cancer cell lines, a normal cell line and a normal human liver. We showed that CCT/TRiC activity does not always correlate with its expression level. We therefore quantified CCT/TRiC activity modulators in the cancer cell lines used throughout this study. We showed that Hop/p60 is not solely responsible of increased or decreased CCT/TRiC-mediated folding efficiency. Similarly, we were not able to reveal a correlation between PhLP3 expression level and CCT/TRiC activity in the cell lines where PhLP3 expression was detectable. We therefore quantified the levels of proteins that assist specifically CCT/TRiC, such as prefoldin, or buffers client proteins pool within the cytoplasm such as Hsc/p70. We showed that the higher prefoldin is expressed, the lower the activity of CCT/TRiC is. We also showed that CCT/TRiC activity is the highest in cell line extracts where Hsc/p70 levels are the lowest. These findings strongly suggest that client proteins partition between prefoldin and/or Hsc/p70 and CCT/TRiC. In the presence of high prefoldin and/or Hsc/p70 concentrations, a significant fraction of client proteins are buffered by the latter molecular chaperones and unavailable for binding to CCT/TRiC while when prefoldin and/or Hsc/p70 concentrations are low the fraction of client proteins bound to CCT/TRiC increases. Thus, variations in the expression levels of prefoldin and/or Hsc/p70 modulate the availability of client protein for CCT/TRiC. This is illustrated by the finding that immuno-depletion of Hsc/p70 cytosolic pool leads to a depletion of sequestered client proteins and a significant decrease in steady state CCT/TRiC activity. The finding that the number of CCT/TRiC and prefoldin particles within the cytosol of cancer cell lines do not differ by over one order of magnitude indicate either that the folding machinery has evolved in such a way that prefoldin and/or Hsc/p70 function in conjunction with CCT or that the maximum amount of client polypeptides the folding machinery can store/buffer and the cell can accommodate is of the same order of magnitude than that of CCT/TRiC particles. This is in particular true in the case the pool of polypeptides associated to prefoldin and/or Hsc/p70 within the cytoplasm represents the sole source of CCT/TRiC client proteins.

Overall, our study reveals a functional interplay between molecular chaperones that might account for a precise modulation of CCT/TRiC activity in cell proliferation through changes in the cellular levels of prefoldin and/or Hsc/p70. Our observations also demonstrate that the availability of client protein for CCT/TRiC is the limiting step that modulates CCT/TRiC activity. Indeed, increased prefoldin and/or Hsc/p70 intracellular concentrations leads to decreased amounts of free client proteins and counteract CCT/TRiC activity. Similarly, an increase of the amount of client protein concentrations at a given prefoldin and/or Hsc/p70 concentration yields free client proteins and restores CCT/TRiC activity. Thus, a tripartite interplay between CCT/TRiC, prefoldin and/or Hsc/p70, on the one hand, client proteins, on the other, appears critical for modulating cell proliferation, in particular in a context where the cytoskeletal and onco-proteins that are critical for cell proliferation and viability are amongst the major clients of CCT/TRiC.

The Hsc/p70 co-chaperones from the Hsp40 family modulate the interaction of Hsc/p70 with its client proteins [37]. We therefore quantified the expression level of one of the major inducible Hsp40 family members [38] in the cancer cell lines used throughout this study. DNAJB1 expression level is the highest in MIA-PaCa-2, MDA-MB-231 and HT29 cells (not shown). This observation is consistent with a higher turnover of Hsc/p70-bound client proteins and the subsequent above average CCT/TRiC activity measured for these extracts. However, DNAJB1 expression levels were relatively modest in two other cell lines (HCT15 and PC-3) exhibiting above average CCT/TRiC activity. Thus, further analysis of the variation of the expression level of other members of the Hsp40 family might allow a better understanding of the interplay between Hsc/p70 and CCT/TRiC in client protein folding.

Further quantification and comparison of the expression levels of other molecular chaperones within cancer and normal cell lines/tissues using the approach we describe here or a global proteomic approach should allow defining the full molecular chaperones network involved in cell proliferation and tumor genesis and the role and importance of CCT/TRiC assisted folding within this network.

## Supporting Information

Figure S1 **Control β-actin folding reactions.** The images recorded using a phosphorimager for [35S]-labeled actin refolding reactions in folding buffer, RRL, RRL+DNase I (µg/ml) and RRL immunodepleted for CCT/TriC resolved on a native 6% polyacrylamide gel are shown.
(TIF)

Figure S2 **Amounts of CCT/TriC, PhLP3, Hop/p60 and PFD.** The averaged concentrations of CCT/TriC, Hop/p60, PhLP3 and PFD in the different cell lines used throughout this study were divided by those in HNCL yielding normalized amounts of (A) CCT/TriC, (C) PhLP3, (E) Hop/p60 and (G) PFD. The amounts of (B) CCT/TriC, (D) PhLP3, (F) Hop/p60 and (H) PFD per cell were determined by dividing their respective concentrations in mg/ml by the number of cells used to prepare the extracts.
(TIF)

Table S1 **Number of active particles of CCT/TRiC and PFD per cell.**
(TIF)

Table S2 **CCT/TRiC activity per cell.**
(TIF)
