# Supplementary figures and images for: The Cytosolic Chaperonin CCT/TRiC and Cancer Cell Proliferation
Source: PLoS One. 2013 Apr 16;8(4):e60895. doi: 10.1371/journal.pone.0060895 (PMC3628893; doi:10.1371/journal.pone.0060895)

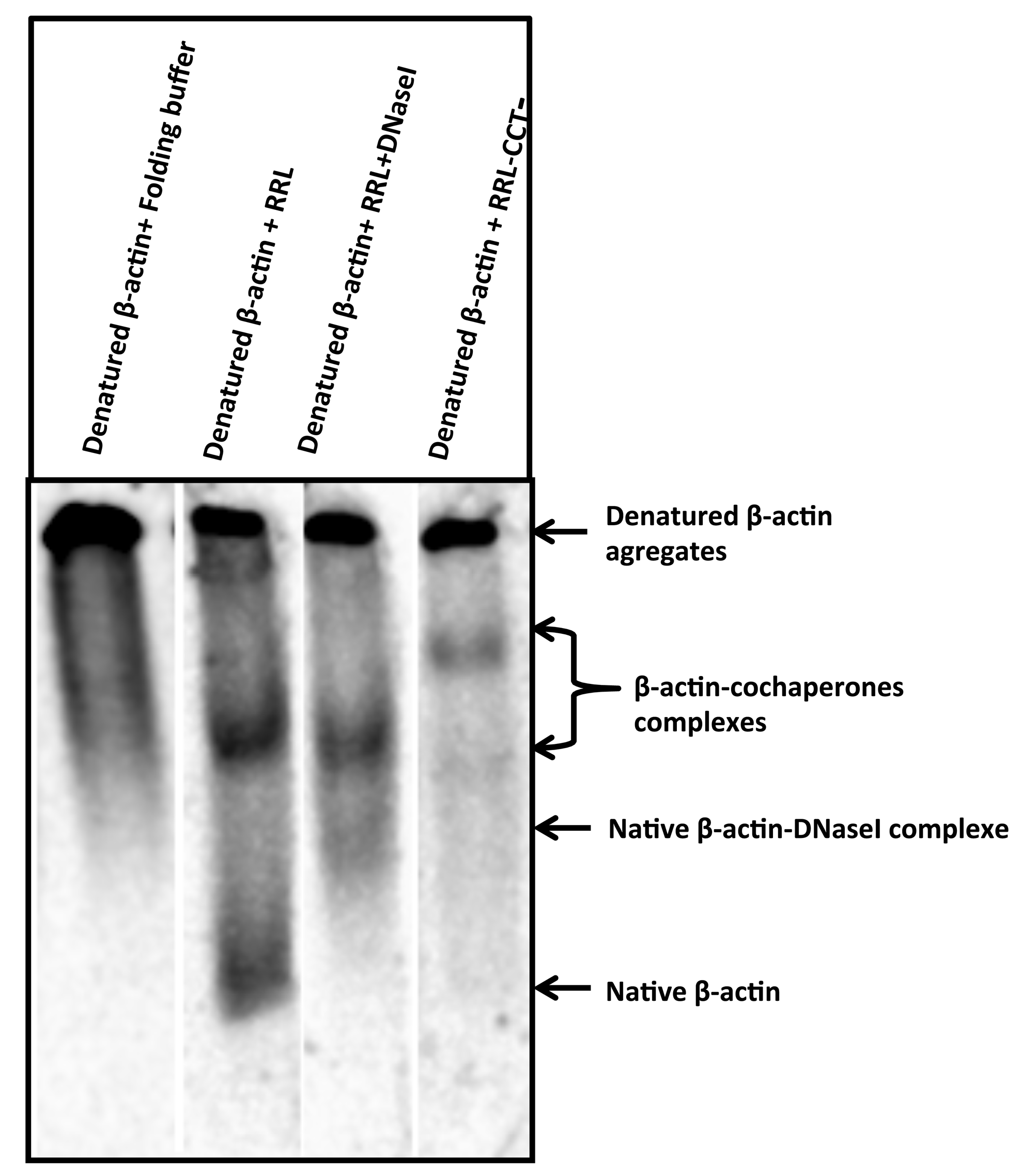

Supplement: Figure S1 — Control β-actin folding reactions. The images recorded using a phosphorimager for [35S]-labeled actin refolding reactions in folding buffer, RRL, RRL+DNase I (µg/ml) and RRL immunodepleted for CCT/TriC resolved on a native 6% polyacrylamide gel are shown. (TIF) [file pone.0060895.s001.tif]

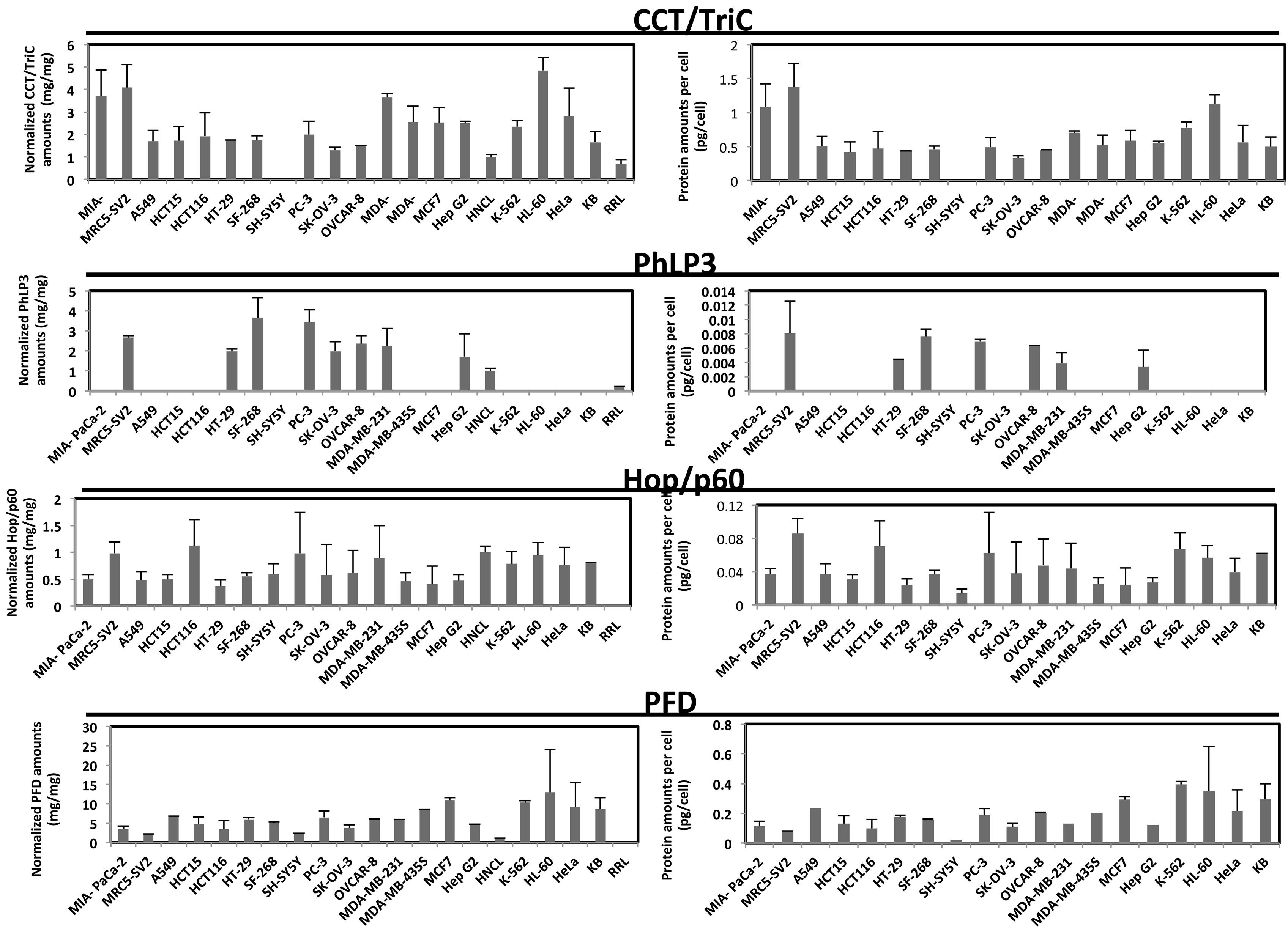

Supplement: Figure S2 — Amounts of CCT/TriC, PhLP3, Hop/p60 and PFD. The averaged concentrations of CCT/TriC, Hop/p60, PhLP3 and PFD in the different cell lines used throughout this study were divided by those in HNCL yielding normalized amounts of (A) CCT/TriC, (C) PhLP3, (E) Hop/p60 and (G) PFD. The amounts of (B) CCT/TriC, (D) PhLP3, (F) Hop/p60 and (H) PFD per cell were determined by dividing their respective concentrations in mg/ml by the number of cells used to prepare the extracts. (TIF) [file pone.0060895.s002.tif]

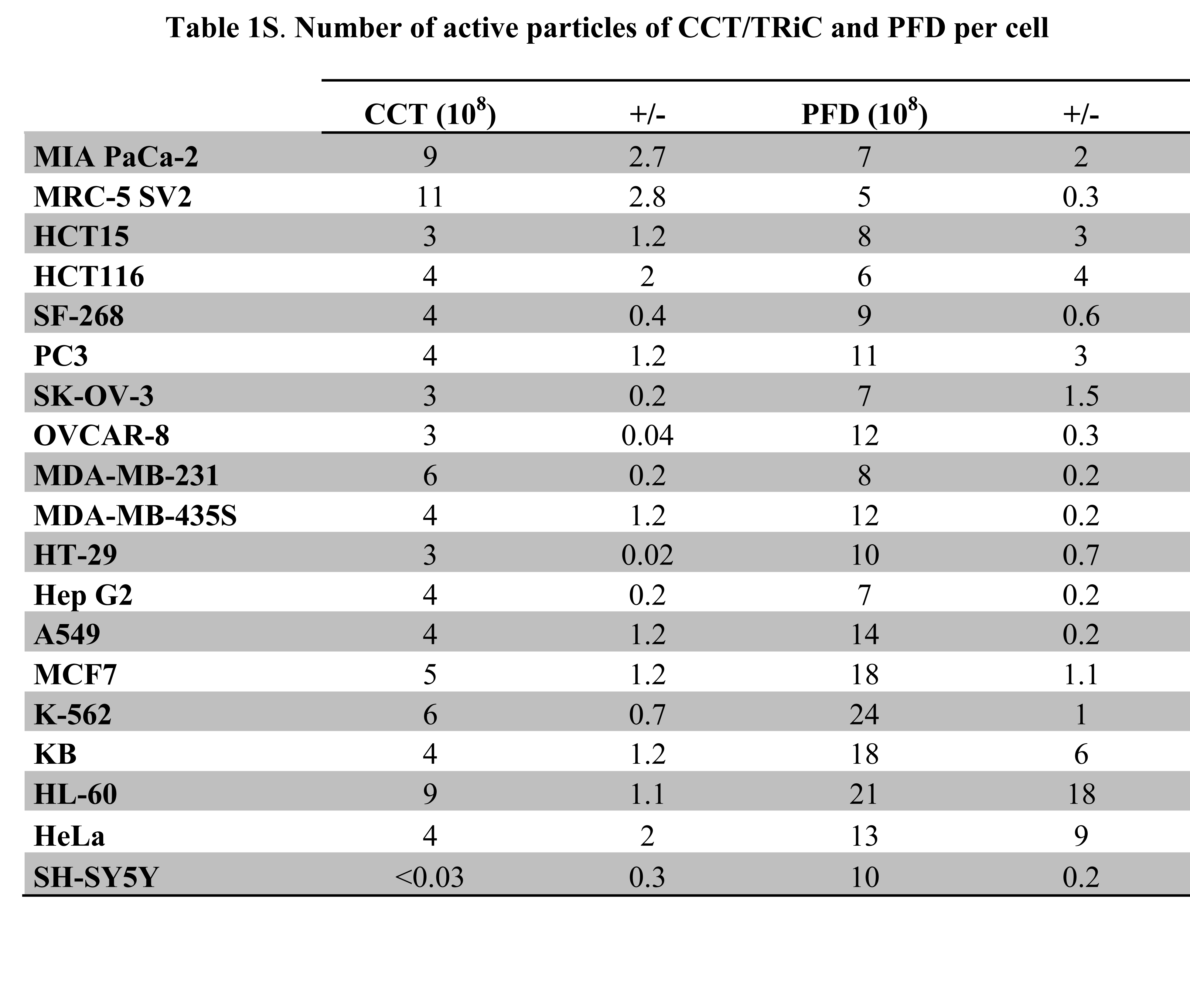

Supplement: Table S1 — Number of active particles of CCT/TRiC and PFD per cell. (TIF) [file pone.0060895.s003.tif]

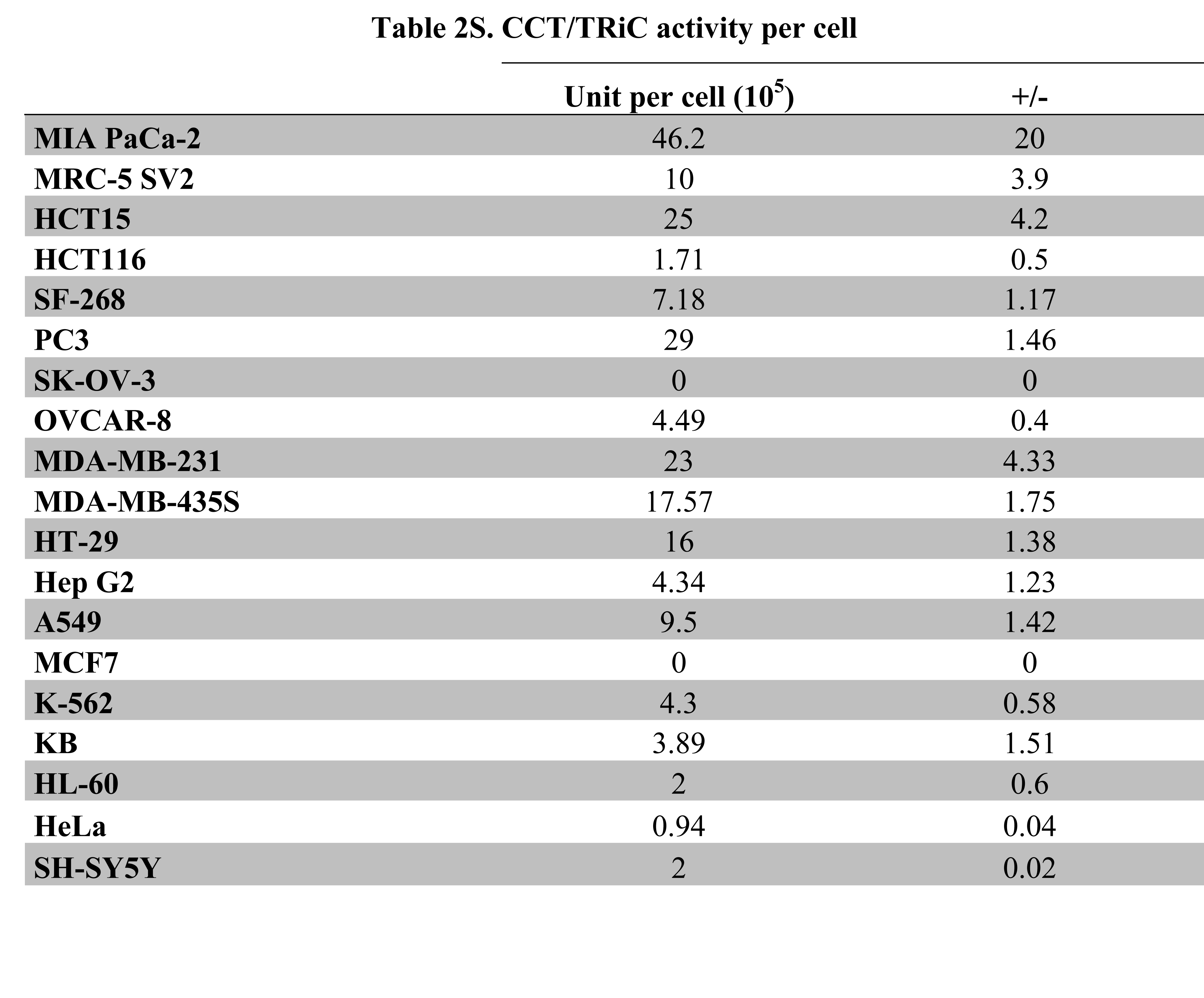

Supplement: Table S2 — CCT/TRiC activity per cell. (TIF) [file pone.0060895.s004.tif]
